# Cholic Acid Induces a *Cftr* Dependent Biliary Secretion and Liver Growth Response in Mice

**DOI:** 10.1371/journal.pone.0117599

**Published:** 2015-02-13

**Authors:** Frank A. J. A. Bodewes, Marcel J. Bijvelds, Willemien de Vries, Juul F. W. Baller, Annette S. H. Gouw, Hugo R. de Jonge, Henkjan J. Verkade

**Affiliations:** 1 Department of Pediatrics, University of Groningen, Beatrix Children’s Hospital—University Medical Center, Groningen, The Netherlands; 2 Department of Gastroenterology & Hepatology, Erasmus University Medical Center, Rotterdam, The Netherlands; 3 Department of Pathology, University Medical Center, Groningen, The Netherlands; CIMA. University of Navarra, SPAIN

## Abstract

The cause of Cystic fibrosis liver disease (CFLD), is unknown. It is well recognized that hepatic exposure to hydrophobic bile salts is associated with the development of liver disease. For this reason, we hypothesize that, CFTR dependent variations, in the hepatic handling of hydrophobic bile salts, are related to the development CFLD. To test our hypothesis we studied, in *Cftr^-/-^* and control mice, bile production, bile composition and liver pathology, in normal feeding condition and during cholate exposure, either acute (intravenous) or chronic (three weeks via the diet). In *Cftr^-/-^* and control mice the basal bile production was comparable. Intravenous taurocholate increased bile production to the same extent in *Cftr^-/-^* and control mice. However, chronic cholate exposure increased the bile flow significantly less in *Cftr^-/-^* mice than in controls, together with significantly higher biliary bile salt concentration in *Cftr^-/-^* mice. Prolonged cholate exposure, however, did not induce CFLD like pathology in *Cftr^-/-^* mice. Chronic cholate exposure did induce a significant increase in liver mass in controls that was absent in *Cftr^-/-^* mice. Chronic cholate administration induces a cystic fibrosis-specific hepatobiliary phenotype, including changes in bile composition. These changes could not be associated with CFLD like pathological changes in CF mouse livers. However, chronic cholate administration induces liver growth in controls that is absent in *Cftr^-/-^* mice. Our findings point to an impaired adaptive homeotrophic liver response to prolonged hydrophobic bile salt exposure in CF conditions.

## Introduction

Cystic fibrosis (CF) is caused by mutations in the CFTR gene [1,2]. Cystic fibrosis liver disease (CFLD) develops in 5–10% of cystic fibrosis patients [3]. It is a serious complication of CF [4,5]. CFLD is characterized by cirrhosis and patients often present with splenomegaly, hypersplenism and complications of portal hypertension, including variceal bleeding and ascites [6,7]. Although the synthesis and excretory functions of the liver are usually spared, liver transplantation can be indicated in some CFLD patients [8]. Despite major advances in basic knowledge about cystic fibrosis, the pathogenesis of CFLD is still unknown.

In the liver, CFTR is expressed exclusively at the apical membrane of the cholangiocytes lining the bile ducts [9,10]. In cholangiocytes, CFTR facilitates the water secretion and the pH of bile [11,12]. Consequently, loss of CFTR function may increase the concentration of the potentially cytotoxic bile salts in bile, leading to damage and occlusion of small bile ducts and, ultimately, obstructive biliary cirrhosis [13–15].

CF mice may show minor gallbladder and liver abnormalities that, spontaneously, do not lead to loss of liver function or CFLD like pathology [16–19]. Compared to humans, mice produce bile with relatively hydrophilic bile salts [20]. Hydrophilic bile salts are considered less cytotoxic than hydrophobic ones, which have been associated with the development of liver disease [21,22]. The hydrophilic profile of murine bile could prevent or mitigate liver disease in CF mice.

We speculate that, Cftr dependent variations, in the hepatic handling of hydrophobic bile salts, might be related to the development of CFLD. To test this hypothesis in mice, we sought to change the composition of murine bile to one containing more hydrophobic bile salts. Therefore, we challenged CF mice, acutely and chronically, with cholic acid (CA), a relatively hydrophobic bile salt with strong detergent activity, and monitored effects on bile production, bile composition, hepatic gene expression, serum alanine transaminase (ALT) levels, liver weight and liver histology [22].

## Materials and Methods

### Animals

We used C57Bl/6;129 *Cftr* ^*-/-*tm1CAM^ mice and *Cftr* ^+/+tm1CAM^ littermate controls. In one additional experiment, we use homozygous F508del *Cftr* mice (*Cftr* ^tm1EUR^) mice and littermate controls. All mice were bred and accommodated at the Animal Experimental Center of the Erasmus Medical Center in Rotterdam, The Netherlands [16]. Mice were housed in a light-controlled (lights on 6 AM to 6 PM) and temperature-controlled (21°C) facility, and had free access to tap water and a semi-synthetic diet (SRM-A; Hope Farms BV Woerden, The Netherlands) from the time of weaning. All experiments were performed with mice of 10–20 weeks of age. Group size varied per experiment, based on breeding success. We used a minimum number of five mice per experimental group. Experimental protocols were approved by the Ethical Committee for Animal Experiments of Erasmus MC. All surgery was performed under sodium pentobarbital anesthesia, and all efforts were made to minimize suffering.

### Experimental procedures

To test our hypothesis we used a murine gall bladder cannulation model [23,24]. Bile was collected after surgical ligation of the common bile duct and cannulation of the gall bladder using polyethylene tubing under intraperitoneal anesthesia with hypnorm (fentanyl/fluanisone 1 μl/g BW) and diazepam (10 μg/g BW). The animals were placed in a temperature and humidity controlled incubator. Bile secretions were collected in 15 or 30 minute fractions. Bile flow rate was assessed gravimetrically, assuming a density of 1g/ml. [23,25].

We first applied acute bile salt infusion, as previously described [25]. After gall bladder cannulation, we infused taurine-conjugated cholate (TCA) (43 mM dissolved in phosphate-buffered saline, pH 7.4) via the jugular vein using an infusion pump. The bile salt dosage was increased every 30 minutes in a stepwise manner (dosage steps 0, 150, 300, 450 and 600 nmol.min^-1^). Bile was collected in 15 minutes fractions.

Different groups of *Cftr* ^*-/-*^ and control mice were either fed the control diet, composed of standard chow or the bile salt diet, composed of standard chow enriched with cholate (CA 0.5% wt/wt), for three weeks. An additional group of *Cftr* ^*-/-*^ and control mice were fed the CA enriched diet for three months. After gallbladder cannulation as described above, bile production was determined by bile collection for 30 minutes. The bile was collected for analysis. Mice were then sacrificed by obtaining a large blood sample via cardiac puncture, followed by cervical dislocation. The liver was excised and weighed, after which samples were either immersed in neutral buffered formalin or stored in liquid nitrogen.

### Analytical techniques

Biliary bile salt concentrations were determined by enzymatic fluorometric assay [26]. Lipids were extracted from the bile [27]. The phospholipid concentrations were determined using a spectrophotometric assay [28]. Biliary bile salt composition was determined by capillary gas chromatography [29]. The hydrophobicity of bile salts in bile was calculated according to the Heuman index, based on the fractional contribution of the major bile salt species [22]. ALT was determined in plasma samples.

RNA was isolated from whole liver for quantitative real time polymerase chain reactions (qRT-PCR) performed on a 7900HT Fast Real-Time PCR system (Applied Biosystems).

For histological analysis of the liver samples, hematoxylin/eosin staining was applied on paraffin embedded sections. To quantify mitotic active cells we used Ki-67 immunostaining using a rabbit anti Ki-67p monoclonal antibody (Novocastra Laboratories Ltd., Newcastle upon Tyne, UK) [30]. An experienced hepato-pathologist (A.S.H.G.) assessed the liver samples in a blinded fashion. Histology of the liver parenchyma and portal tracts were evaluated separately for inflammation, fibrosis, and Ki-67 mitotic activity. The score was performed in a semi-quantitative approach on a scale from 0 to 3 (0: absent, 1: sporadic, 2: regular, 3: frequent) [31].

### Statistical analysis

The statistical analysis was performed using SPSS version 18.0 for Windows (SPSS Inc., Chicago, IL). All numerical results are reported as means ± SEM. The ordinal histology results are reported as medians and range. Differences between study groups were evaluated using the Mann-Whitney U test. The level of significance was set at a P value of less than 0.05.

## Results

### Bile production and bile salt secretion after interruption of the enterohepatic circulation

**Fig 1:**
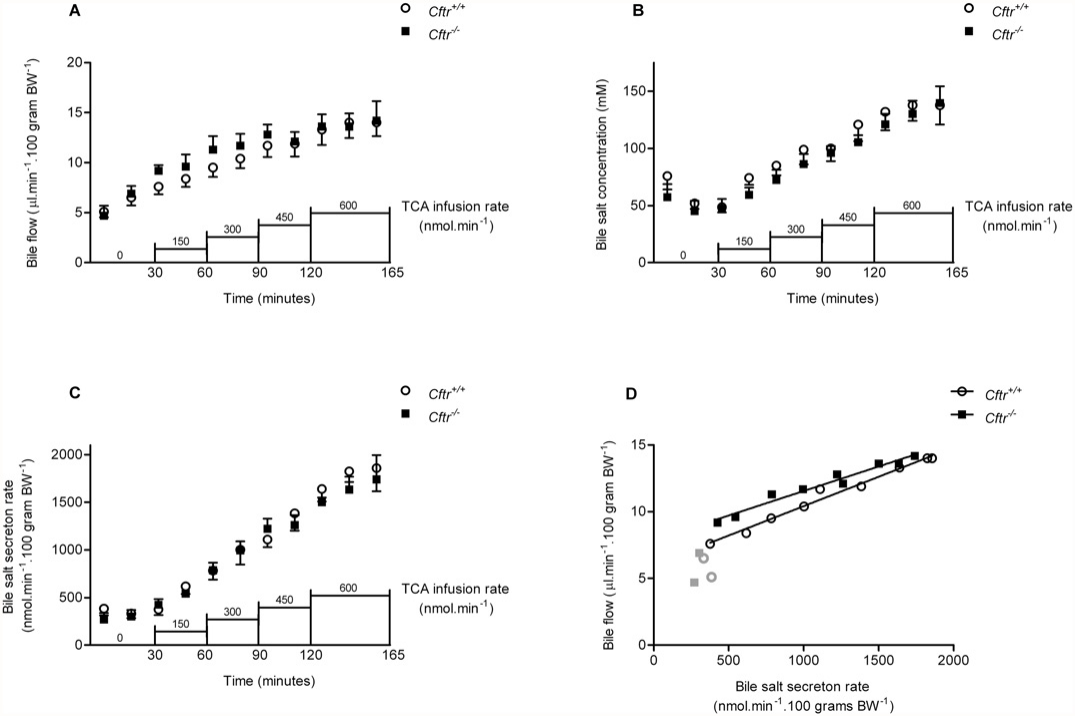
Biliary parameters during intravenous taurocholic acid (TCA) administration. Biliary bile flow (A), bile salt concentration (B), bile salt secretion rate (C) and relationship between bile salt secretion rate and bile flow (D) in Cftr knockout mice (*Cftr* ^*—/*^) and control littermates (*Cftr* ^*+/+*^) during intravenous infusion with TCA in stepwise increasing dosage of. The gray symbols in (D) represent baseline values before the start of TCA infusion. Data are presented as means ± SEM of N = 5–7 mice per group. There was no significant difference between *Cftr* ^*-/-*^ and *Cftr* ^*+/+*^ mice, at any of the individual time points, for bile flow, bile salt concentration and bile salt secretion rate (regression lines:. *Cftr* ^*-/-*^: y = 0.0044x + 7.9 vs. *Cftr* ^*+/+*^: y = 0.0037x + 6.0).

Bile production, assessed over 30 minutes immediately after interruption of the enterohepatic circulation, did not differ significantly between *Cftr* ^*-/-*^ mice and controls (mean of 2 15 min points in first 30 minutes: 6.3±0.7 vs. 5.8±0.5 μl.min^-1^.100 g-^1^, respectively; Fig. 1A). Bile salt concentrations and bile salt secretion rates were also similar between *Cftr* ^*-/-*^ mice and controls under these untreated conditions (Fig. 1B and C).

### Bile production and bile salt secretion during acute IV taurocholic acid (TCA) administration

Administration of TCA (IV) dose-dependently increased bile production (Fig. 1A). The dose-response relationship was similar for *Cftr* ^*-/-*^ mice and controls, indicating that bile production was not impaired in CF mice. Also, a similar, concomitant dose-dependent increase in bile salt concentrations and secretion rates was observed during TCA infusion, in *Cftr* ^*-/-*^ mice and controls (Fig. 1B and C). The bile salt secretion rate was linearly correlated with the bile flow (Fig. 1D), with similar slopes in *Cftr* ^*-/-*^ and control mice, indicating similar magnitudes of bile salt dependent bile flow. These observations strongly suggested that the bile flow in mice, both under basal conditions and after acute IV TCA administration, is predominantly generated via Cftr independent mechanisms.

### Bile production and bile salt composition during chronic cholic acid (CA) feeding

**Fig 2:**
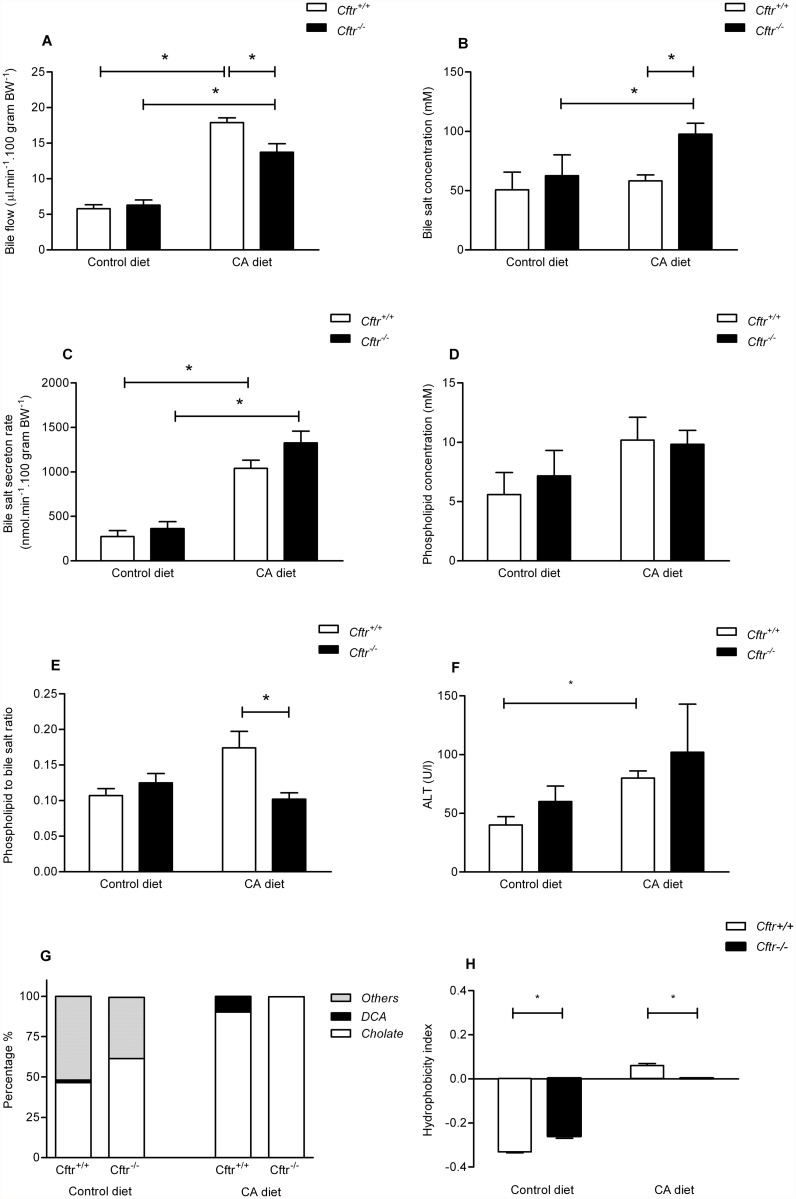
Biliary parameters and bile composition of Cftr knockout mice (Cftr^-/-^) and controls, fed a control diet or a cholic acid (CA) containing diet for 3 weeks. Biliary bile flow (A), bile salt concentration (B), bile salt secretion rate (C), phospholipid concentration (D), phospholipid-to-bile salt ratio (E), serum ALT levels (F), percent contribution to total biliary bile salts of cholate, deoxycholate and others (chenodeoxycholate, ursodeoxcholate, α-muricholate and β-muricholate) (G), and, Heuman index of biliary bile salts representing the hydrophobicity (H), in Cft*r* knockout mice (*Cftr* ^*-/-*^) and control littermates (*Cftr* ^*+/+*^) after a control or 0.5%-CA (wt/wt) chow diet for three weeks. Data are presented as means ± SEM or percentage (panel G) of N = 5–7 mice per group. *P-value<0.05.

Feeding a CA containing diet for three weeks increased bile flow in *Cftr* ^*-/-*^ mice and controls (Fig. 2A). However, the increase was significantly more pronounced in controls than in *Cftr* ^*-/-*^ mice. The CA diet increased the biliary bile salt concentration in *Cftr* ^*-/-*^ mice but not in controls (Fig. 2B). Chronic CA feeding increased the bile salt secretion rate in *Cftr* ^*-/-*^ mice and controls. However, the bile salt secretion rate, during CA diet was not significantly different between *Cftr* ^*-/-*^ mice and controls (Fig. 2C).

To assess cytotoxicity, we measured the biliary phospholipid concentration and calculated the phospholipid-to-bile salt ratio. During regular chow feeding (control diet), biliary phospholipid concentrations were similar in *Cftr* ^*-/-*^ mice and controls (Fig. 2D), as were the phospholipid to bile salt ratios (Fig. 2E). CA feeding seemed to increase phospholipid concentrations, both in *Cftr* ^*-/-*^ and controls, but the differences between the diets did not reach statistical significance (Fig. 2D). Also, the phospholipid concentration did not significantly differ between *Cftr* ^*-/-*^ and control mice on either diet. CA administration increased the phospholipid to bile salt ratio only in control mice (Fig. 2E). On control diet, serum ALT levels were similar in *Cftr* ^*-/-*^ and control mice (Fig. 2F). CA feeding slightly, but significantly, increased serum ALT in control mice suggestive for minor hepatocyte injury. Also ALT levels also increased in *Cftr* ^*-/-*^ mice upon the CA diet, but this was statistically not significant.

As anticipated, the biliary bile salt compositions in *Cftr* ^*-/-*^ and control mice were relatively hydrophilic (i.e. negative Heuman indices), upon feeding the control diet (Fig. 2H). Even at these control conditions, however, the hydrophobicity index was significantly higher in *Cftr* ^*-/-*^ mice compared with controls (-0.33 vs. -0.26; respectively, P<0.01). The higher hydrophobicity index could be attributed mainly to a higher fractional CA content in *Cftr* ^*-/-*^ mice (Fig. 2G). The CA diet markedly increased the fractional contribution of CA to the total biliary bile salt pool in both groups. In control mice, the CA contribution increased to ~90% whereas it was even close to 100% in *Cftr* ^*-/-*^ mice. In the control mice, the remaining 10% consisted predominantly of the secondary bile salt deoxycholic acid. Since DCA is a more hydrophobic bile salt than CA, the hydrophobicity index of the bile, after prolonged CA exposure, was higher in controls than in *Cftr* ^*-/-*^ mice (0.06 vs. 0.00, respectively, P<0.01, Fig. 2H).

**Fig 3:**
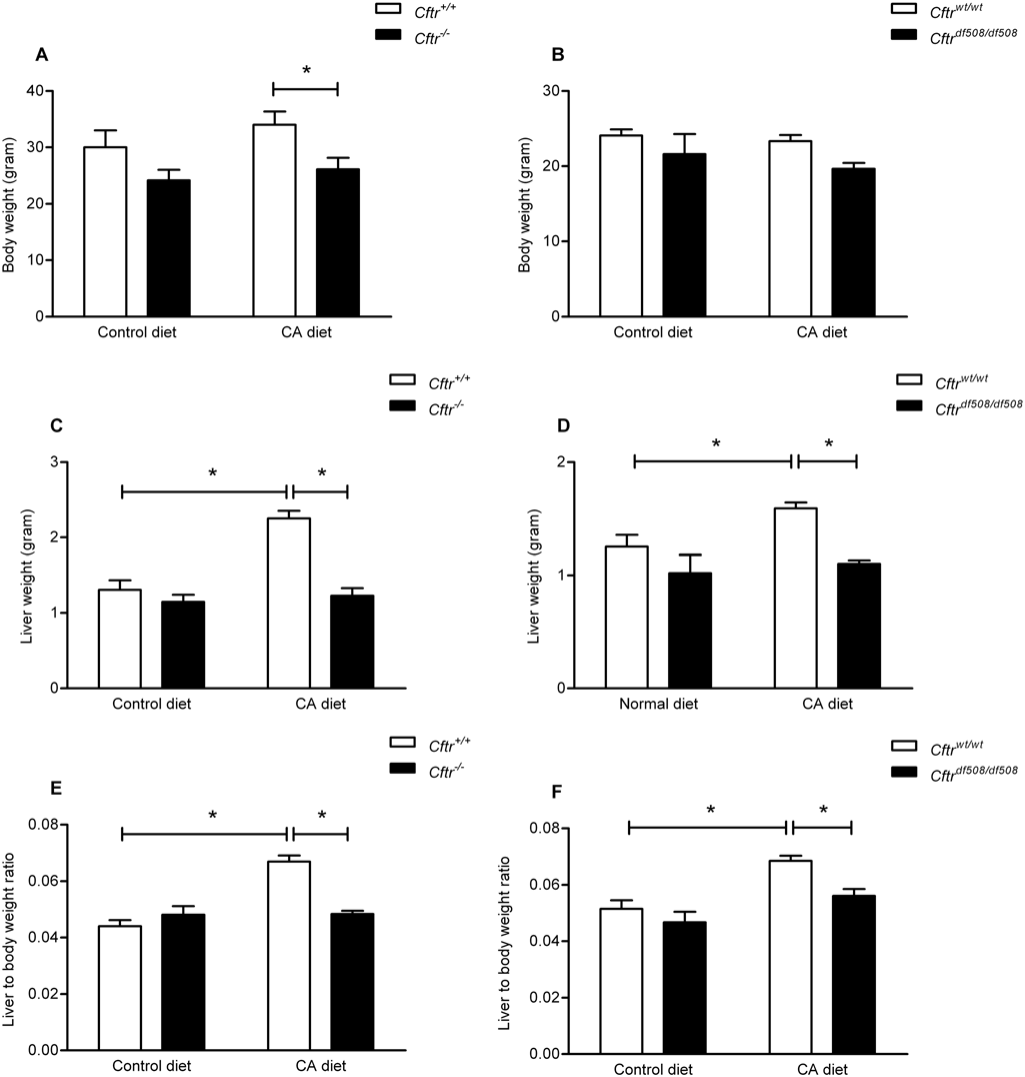
Hepatic Cyp7a1 mRNA expression Cftr knockout mice (Cftr^-/-^) and controls, fed a control diet or a cholic acid (CA) containing diet for 3 weeks. Hepatic Cyp7a1 mRNA expression after a control (A) or 0.5%-CA (wt/wt) chow diet (B) for three weeks. Cyp7a1 mRNA levels were normalized to a housekeeping gene (β-actin) in *Cftr* knockout mice (*Cftr* ^*-/-*^) and control littermates (*Cftr* ^*+/+*^). Data are presented as means ± SEM of N = 5–7 mice per group. *P-value<0.05.

Hydrophobic bile salts are regarded as strong ligands for the nuclear receptor Fxr, which is, among other functions, also involved in bile acid synthesis regulation. The observed significant difference in DCA proportion upon CA treatment could correspond with differences in bile synthesis rates between the genotypes. To address this possibility, we determined the mRNA expression of hepatic *Cyp7a1*, encoding the rate-limiting enzyme in bile salt synthesis from cholesterol. During normal diet, the hepatic expression of Cyp7a1 was similar in *Cftr* ^*-/-*^ and control mice. After prolonged CA exposure, however, the Cyp7a1 expression was completely suppressed in control mice, but unaffected in *Cftr* ^*-/-*^ mice (Fig. 3A and B).

### Cftr dependent effects of chronic CA exposure on liver weight

**Fig 4:**
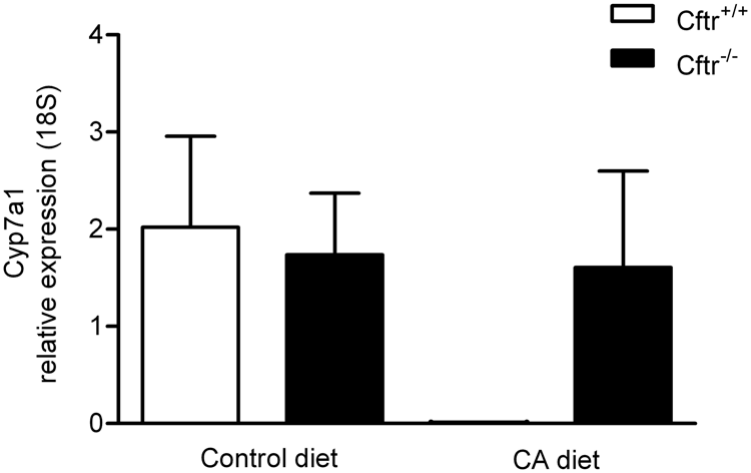
Body and liver weights in Cftr knockout, ΔF508 Cftr and control mice, fed a control diet or a cholic acid (CA) containing diet for 3 weeks. Body weight (A, B), absolute liver weight (C,D) and relative liver weight reported as a percentage of the body weight (E, F) in *Cftr* knockout mice (*Cftr* ^*-/-*^, panels A. C and E) or ΔF508 *Cftr* mice (*Cftr* ^tm1EUR^, panels B, D and F) and their respective control littermates (*Cftr* ^*+/+*^), after a control or 0.5%-CA (wt/wt) chow diet for three weeks. Data are presented as means ± SEM of N = 5–7 mice per group. *P-value<0.05.

In accordance with earlier reports, *Cftr* ^*-/-*^ mice tended to a lower body weight as littermate controls (Fig. 4A) [32]. CA feeding did not markedly affect body weight, but nevertheless, the difference between the two genotypes now was significant. On control diet, *Cftr* ^*-/-*^ and control mice had similar absolute and relative liver weights (Fig. 4C and 4E). Chronic CA administration increased the absolute and relative liver weight in controls (+52%, p<0.001), but did not affect either in *Cftr* ^*-/-*^ mice (Fig. 4C and 4E). We also observed the absence of CA-induced liver growth response in mice expressing mutant *Cftr* (homozygous F508del *Cftr* mice; *Cftr* ^tm1EUR^), in contrast to their wild type littermates (Fig. 4B, 4D and 4F) [33]. The combined observations indicate that chronic CA exposure induces liver growth, in different mouse strains, but does require functional Cftr expression.

### Cftr dependent effects of chronic CA exposure on liver histology

**Fig 5:**
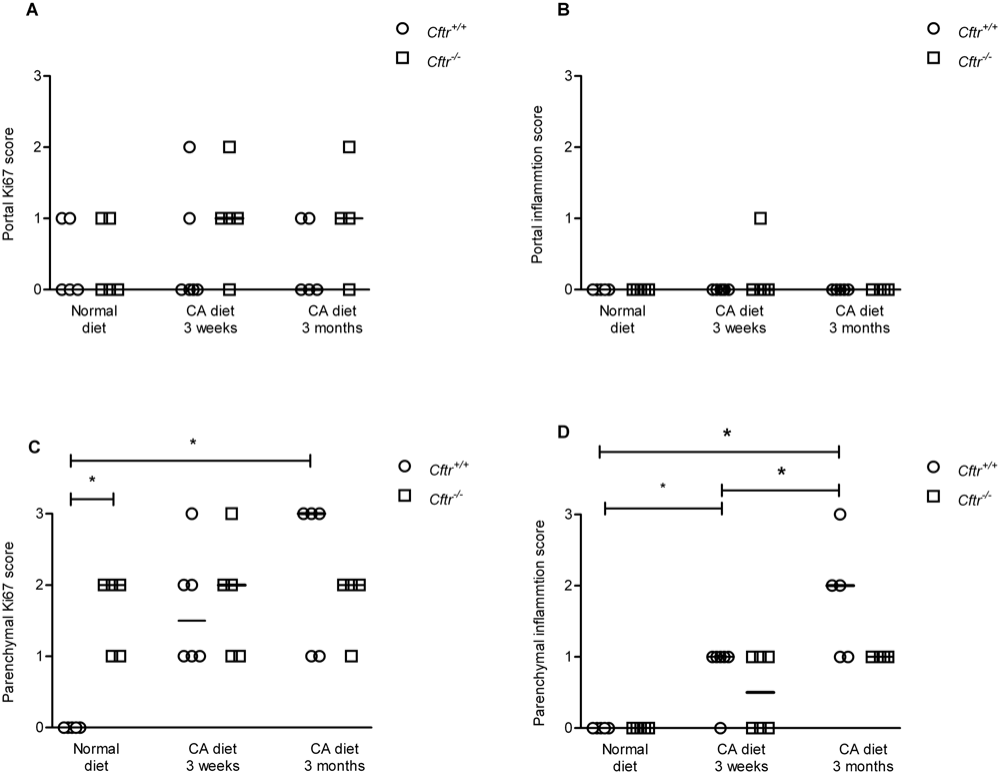
Liver histology in Cftr knockout mice (Cftr^-/-^) and controls, fed a control diet or a cholic acid (CA) containing diet for 3 weeks or for 3 months. Histological evaluation of liver parenchymal inflammation (A), parenchymal Ki67 activity (B), portal inflammation (C), and portal Ki67 activity (D), in Cftr knockout mice (*Cftr* ^*-/-*^) and control littermates (*Cftr* ^*+/+*^) after a control diet, a 0.5%-CA (wt/wt) chow diet for three weeks, or a 0.5%-CA (wt/wt) chow diet for three months. Histology was scored in a blinded and semi-quantitative fashion using an ordinal scale (0: absent; 1: sporadic; 2: regular; 3: frequent). All results are presented individually per mouse; the horizontal black line represents the median of N = 5–7 mice per group. *P-value<0.05.

**Fig 6:**
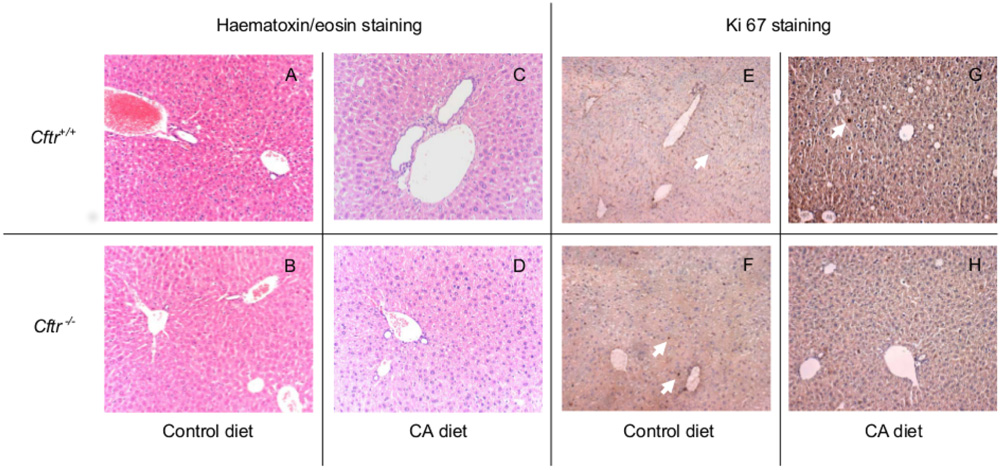
Liver histology of Cftr knockout mice (Cftr^-/-^) and controls, fed a control diet or a cholic acid (CA) containing diet for 3 weeks. Liver histology (magnification 40x) of *Cftr* knockout mice (*Cftr* ^*-/-*^) and control littermates (*Cftr* ^*+/+*^) after a control or 0.5%-CA containing diet for three weeks. Liver slices with HE staining (panels: A-D), or Ki67 staining on HE staining background (panels: E-H). The white arrows, pointing at Ki67 positive cells indicate active cell proliferation in parenchymal and portal areas.

On control diet, parenchymal cell proliferation was enhanced in *Cftr* ^*-/-*^ mice, relative to controls (Fig. 5C), but no signs of parenchymal inflammation were evident. Chronic CA administration increased parenchymal mitotic activity in controls to a level similar to that found in *Cftr* ^*-/-*^ mice on either regular chow or after chronic CA exposure. Concomitantly, three week CA feeding significantly increased the parenchymal inflammation score in controls (Fig. 5D). Prolonged CA exposure (three months) further increased parenchymal mitotic activity and inflammation, particularly observed in controls. CA feeding did not generate pathological changes in the portal tract areas of *Cftr* ^*-/-*^ or control mice (Fig. 5A and C). Fig. 6 shows representative images of liver histology after control and three weeks CA diet (panel A-D: HE staining and panel E-H: Ki67 staining).

## Discussion

In *Cftr* ^*-/-*^ mice, both acute and chronic hydrophobic bile salt administration markedly increased biliary bile salt production and flow rates. However, *Cftr* ^*-/-*^ mice showed a reduced capacity to adapt to chronic hydrophobic bile salt exposure, compared with control mice. Although biliary bile salt production was similar in *Cftr* ^*-/-*^ and control mice, the increase in bile flow was significantly less pronounced in the former. The reduced capacity, of the *Cftr* ^*-/-*^ mice, to increase bile flow, during prolonged hydrophobic bile salt administration, resulted in significantly higher bile salt concentrations in bile. Interestingly, acute intravenous bile salt administration did not induce similar differences between *Cftr* ^*-/-*^ and control mice, indicating that only adaptations to prolonged hydrophobic bile salt exposure are perturbed in CF.

Prolonged CA exposure did not result in CFLD like, histopathology in *Cftr* ^*-/-*^ mice, despite their higher biliary bile salt concentration and lower bile salt to phospholipids ratio. These results make it unlikely that hydrophobic bile salt cytotoxicity underlies the pathogenesis of CFLD. During normal feeding conditions, the hydrophobicity index of bile of *Cftr* ^-/-^ mice was slightly higher than in control mice, due to an increased proportion of cholate. In contrast, however, the hydrophobicity index of bile was lower than in controls after prolonged CA exposure. The lower hydrophobicity in *Cftr* ^*-/-*^ mice, after prolonged CA exposure, was due to a higher contribution of the hydrophobic secondary bile salt DCA in the control mice. DCA is exclusively formed from CA by the intestinal bacterial microflora [34–36]. From these findings we suggest that *Cftr* dependent alterations in the intestinal, bacterial biotransformation of bile salts are responsible for the different biliary bile salt composition and hydrophobicity in CF mice conditions.

CF mouse models, in general, do not display histopathological signs of liver disease [37]. As an exception, however, Durie et al. reported the development of spontaneous liver disease upon ageing in the C57BL/6J *Cftr* ^*-/-*tm1Unc^ CF mice [17]. The *Cftr* ^*-/-*tm1Unc^ mice used by Durie et al. have a different genetic background than the mice used in our present study. The differences between the mouse strains are compatible with the concept genetic modifiers affect the hepatobiliary phenotype of CF. Considering the potential effects of intestinal microflora on bile salt composition, environmental factors may additionally play a role [36].

Chronic CA exposure significantly increased liver mass in control mice. Interestingly, CA induced liver growth was absent in CF mice. It was described previously that hydrophobic bile salts can induce liver growth in mice. Huang et al. reported that CA feeding (0.2%) for five days increased the relative liver weight by approximately 30% in wild type mice [38]. Song et al. reported an 8% increase in relative liver weight in C57BL/6 mice fed a CA enriched diet for one week [39]. These scientists also reported that DCA 0.3% for one week induce a 32% increase in relative liver weight suggesting that the effect of the more hydrophobic bile salt DCA on liver growth was even more pronounced than that of CA.

It has been established that bile salt induced homeotrophic liver growth is regulated via the nuclear receptor Fxr [40], either in the liver or in the intestine [41]. Our finding that liver growth was absent in CA-fed Cftr^-/-^ and Cftr^ΔF508/ΔF508^ mice suggested that FXR signaling is perturbed in CF mice. This suggestion was supported by differences in *Cyp7a1* mRNA expression between *Cftr* ^*-/-*^ and control mice after prolonged CA exposure. Hepatic *Cftr* is exclusively expressed in cholangiocytes, what makes *Cftr-Fxr* interference at the level of the liver less likely. The intestinal Fxr stimulated liver proliferation is probably mediated via the induction of the expression of Fgf15 in the intestine [42]. Uriarte et al.recently reported that liver growth elicited by CA feeding was significantly diminished in *Fgf15* ^*-/-*^ mice [43]. Since Fgf15 expression is regulated by intestinal Fxr, these findings are compatible with a role for the intestinal Fxr-Fgf15 axis in liver growth induction after hydrophobic bile salt exposure. In accordance with this concept, Debray et al. showed that Fgf15 expression in *Cftr* ^*-/-*^ mice was low at the level of the ileum, suggesting altered Fxr bile salt signaling at the level of the intestine in CF conditions [44].

Reactive oxygen species (ROS)-dependent signaling could also play a role in stimulation of hepatocyte proliferation and liver regeneration [45]. Sokol et al. demonstrated that, in human hepatocytes, mitochondria can generate ROS when exposed to hydrophobic bile salts [46]. However, we consider it less likely that this mechanism is involved for the liver growth and hepatocyte proliferation found in our model. As stated above *Cftr* is only expressed in the cholangiocytes and not in hepatocytes. In addition, we determined hepatic heme-oxygenase 1 (HO-1) expressions in our models, as a marker of hepatic oxidative stress. We observed no differences between HO-1 expression between *Cftr* ^*-/-*^ and control mice before or after three week CA exposure (data not shown). These considerations and findings indicate that, at least at the hepatic level, chronic CA exposure, and liver growth are unlikely to be related to ROS-dependent signaling or oxidative stress.

In summary, we show that, in mice, *Cftr* plays a role in the hepatic adaptive response to prolonged hydrophobic bile salt exposure. The CF condition is characterized by a reduction in bile salt-induced hepatocyte proliferation, increase in biliary bile salt concentrations and difference in secondary bile salt composition. These changes suggest that the CF condition may be more prone to liver disease due to an impaired liver regeneration and homeotrophic liver growth response upon exposure to hepatotoxic substances.
